# Genetic architecture and regulatory impact on hepatic microRNA expression linked to immune and metabolic traits

**DOI:** 10.1098/rsob.170101

**Published:** 2017-11-08

**Authors:** Siriluck Ponsuksili, Nares Trakooljul, Frieder Hadlich, Fiete Haack, Eduard Murani, Klaus Wimmers

**Affiliations:** 1 Research Unit ‘Functional Genome Analysis’, Leibniz Institute for Farm Animal Biology (FBN), Wilhelm-Stahl-Allee 2, 18196 Dummerstorf, Germany; 2 Research Unit ‘Genomics’, Leibniz Institute for Farm Animal Biology (FBN), Wilhelm-Stahl-Allee 2, 18196 Dummerstorf, Germany; 3 Faculty of Agricultural and Environmental Sciences, University Rostock, 18059 Rostock, Germany

**Keywords:** non-coding RNAs, transcriptome, biomarker, post-transcriptional regulation, expression-QTL, swine

## Abstract

Regulation of microRNA (miRNA) expression contributes to a wide range of target gene expression and phenotypes. The miRNA expression in the liver, the central metabolic organ, was examined in 209 pigs, and integrated with haematological and clinical biomarkers of metabolic and overall health, mRNA-target expression levels and single-nucleotide polymorphism (SNP) genotypes. The expression levels of 426 miRNA species correlated with plasma haematological or biochemical traits (*r*² = |0.19–0.45|, false discovery rate < 5%). Pairs of these miRNAs and their predicted target mRNAs showing expressing levels associated with the identical traits were examined to understand how immune and metabolic traits are affected by miRNA-mediated regulatory networks derived by mapping miRNA abundance as an expression quantitative trait. In total, 221 miRNA-expression-QTL correspond to 164 SNPs and 108 miRNAs, including miR-34a, miR-30e, miR-148-3p, miR-204, miR-181-5p, miR-143-5p and let-7 g that also correlate with the biomarkers. Sixty-one SNPs were simultaneously associated with 29 miRNA and 41 mRNA species. The expression levels of 13 out of 29 miRNA were correlated with one of the biochemical or haematological traits. For example, the expression levels of miR-34a were correlated with serum phosphorus and cholesterin levels; miR-204, miR-15a and miR-16b were correlated with triglyceride. For haematological traits, the expression levels of miR-652 and miR-204 were correlated with the mean corpuscular haemoglobin concentration, and the expression of miR-143 was correlated with plateletcrit. Pleiotropic association analyses revealed genetic links between mRNA and miRNA on SSC6 for miR-34a, SSC9 for miR-708 and SSC14 for miR-652. Our analysis of miRNA and mRNA transcript profiles, their correlation with clinically important plasma parameters of hepatic functions as well as information on their genetic regulation provide novel regulatory networks and potential new biomarkers for immune and metabolic traits.

## 1. Introduction

MicroRNAs (miRNAs) are small endogenous non-coding molecules ranging from 18 to 24 nt that control target transcripts at the post-transcriptional level. miRNAs target mRNA transcripts via basepair complementarity, typically in the 3′ untranslated region [1,2], but may also target the coding sequence [3]. This targeting can induce transcript cleavage, degradation, destabilization and repression of translation, thereby modulating protein levels. It has been recently shown that reduction in target mRNA levels accounts for most of the regulatory, repressive effects of miRNAs [4]. Moreover, miRNA expression profiles have been associated with many complex traits and diseases [5–7]. Coordinated miRNA and mRNA expression in Erhualian and Large White Pigs was analysed in order to contribute to the understanding of breed-specific metabolic characters [8].

Expression-QTL (eQTL) analysis integrates gene expression and genome-wide genotype information to identify genetic variation associated with changes in gene expression. Many eQTL mapping studies reveal genetically regulated mRNA transcripts in various tissues such as liver, brain, muscle, blood and fat [9–14]. eQTL-based analysis provides insight into miRNA regulation [15]; compared with mRNA transcripts, there are relatively few studies of miRNA-eQTL [16–20]. Most studies use miRNA expression profiles from whole blood, cell lines and adipose tissue [17–19]. Though liver is the central metabolic organ, and plays a vital role in maintaining homeostasis and health, as well as regulating nutrient utilization, there is only one report of genetic regulated miRNA expression in the liver [21]. Knowledge of the genetic regulation of liver miRNA expression profiles will provide insight into haematological, biochemical and clinical–chemical biomarkers of hepatic function supporting metabolic homeostasis, innate defence and resilience. Pigs share many similarities in physiology and genome content with humans, and therefore provide an excellent model for medical research, including studies on liver transplantation [22].

The identification of single-nucleotide polymorphisms (SNPs) associated with miRNA abundance in the liver has the potential to aid in the understanding of the role of miRNAs in metabolism and immune processes. We examined the genetic regulation of porcine liver miRNA expression and its consequences on plasma haematological and biochemical traits that are biomarkers of liver function and surrogate traits for immune and metabolic status. Additional links between predicted mRNA targets were identified along with miRNA and mRNA that correlated with the biomarkers.

## 2. Results

### 2.1. Trait-correlated hepatic microRNAs expression

Expression levels of 826 different miRNA species from 209 individuals were used for correlation analysis. At a significance level of FDR < 5%, expression levels of 426 miRNA species correlated with plasma biomarkers of immune and metabolic status. All measurements and descriptions of the traits are shown in electronic supplementary material, table S1.

At a significance level of FDR < 5%, expression levels of 330 miRNA species correlated with at least one of the biochemical traits (albumin, ALB; ammonia nitrogen, NH3; blood urea nitrogen, BUN; total cholesterol, TCHO; triglyceride, TG; glucose, GLU; inorganic phosphorus, IP; creatinine, CREA). In total, 549 correlations between miRNA expression and biochemical traits were found at *r* = |0.19–0.45| (electronic supplementary material, table S2). Table 1 shows 63 miRNA that are listed as porcine miRNAs in miRbase (SSC-miRNA) correlated with various biochemical traits at FDR < 5%.

**Table 1.** Porcine miRNA (SSC-miRNAs of miRbase) showing negative and positive correlation (*r*) with biochemical traits at FDR < 5%.

phenotype	positive correlation of miRNA	negative correlation of miRNA	*r*	*p*-value
ALB	miR-335	miR-146a-5p, miR-20a, miR-21, miR-152	0.19–0.22	4.85 × 10^−3^–1.26 × 10^−3^
BUN	miR-193a-3p, miR-145-5p, miR-29b	miR-18a	0.19–0.27	4.27 × 10^−3^–5.81 × 10^−5^
CREA	miR-130a, miR-34a	miR-193a-5p, miR-125b, miR-92a	0.19–0.26	5.11 × 10^−3^–8.11 × 10^−5^
GLU	miR-143-3p, miR-193a-3p, miR-324, miR-30b-5p, miR-30e-5p, miR-130a, miR-19b, miR-29b	let-7c, let-7d-5p, let-7a, miR-26a, miR-92b-5p	0.19–0.28	4.92 × 10^−3^–4.04 × 10^−5^
IP	miR-885-5p, miR-92a, miR-4332, miR-92b-3p, miR-331-3p, miR-744, miR-92b-5p, miR-4334-5p	miR-34a, miR-194b-5p, miR-20a, miR-15a, miR-146a-5p, miR-152	0.19–0.42	5.08 × 10^−3^–1.08 × 10^−10^
NH3		miR-455-5p, let-7i, miR-497, miR-34a, miR-362, miR-363, miR-181d-5p, miR-22-5p	0.19–0.25	4.28 × 10^−3^–1.63 × 10^−4^
TCHO	miR-1307, miR-744	miR-146a-5p, miR-152, miR-15a, miR-34a	0.19–0.22	4.56 × 10^−3^–1.55 × 10^−3^
TG	miR-106a, miR-92b-5p, miR-92a, miR-4332, miR-222 miR-107, miR-30d, miR-103, miR-744	miR-15a, let-7 g, let-7i, let-7f, miR-199b-3p, miR-10a-5p, miR-199a-3p, miR-148a-3p, miR-20a, miR-195, miR-7, miR-218b, miR-30e-3p, miR-146a-5p miR-21, miR-499-5p, miR-676-3p, miR-148b-3p, miR-29c, miR-98, miR-100, miR-204, miR-27b-3p	0.19–0.33	4.55 × 10^−3^–8.90 × 10^−7^

Haematological traits include white blood cell count (WBC), lymphocyte count (LYM), red blood cell count (RBC), haemoglobin concentration (HGB), haematocrit level (HCT), mean corpuscular volume (MCV), mean corpuscular haemoglobin (MCH), mean corpuscular haemoglobin concentration (MCHC), red distribution width (RDW), platelets (PLT), mean platelet volume (MPV) and plateletcrit (PCT). At a significance threshold of FDR < 5%, we detected 1027 trait-correlated expressions; among them, 350 miRNAs were correlated with at least one haematological trait (electronic supplementary material, table S3). The correlation between expression levels and haematological traits ranged between *r* = |0.19–0.44|. Most transcripts were correlated with erythrocytes and a few with platelets. Eighty SSC–miRNA were correlated with one of the haematological traits with FDR < 5% (table 2).

**Table 2.** Porcine miRNA (SSC-miRNAs of miRbase) showing negative and positive correlation (*r*) with haematological traits at FDR < 5%.

phenotype	positive significant miRNA	negative significant miRNA	*r*	*p*-value
WBC	miR-28-5p	miR-4332, miR-92b-5p, miR-16, miR-145-5p, miR-1307	0.19–0.24	5.22 × 10^−3^–3.57 × 10^−4^
LYM	let-7d-5p, let-7i, miR-146a-5p, miR-15a, miR-27b-3p, miR-204, let-7a, miR-151-3p, miR-421-3p, miR-194b-5p, miR-28-5p, miR-199b-3p, let-7e, miR-215, miR-199a-3p, miR-30e-3p, miR-151-5p, miR-361-5p, miR-98, miR-218b, let-7f, miR-30a-3p, miR-21, miR-182, miR-10a-5p, miR-150, miR-195	miR-30d, miR-4334-5p, miR-22-3p, miR-107, miR-4332, miR-30e-5p, miR-324, miR-103, miR-222, miR-17-5p,miR-335, miR-362, miR-451	0.19–0.32	4.95 × 10^−3^–2.90 × 10^−6^
HCT	miR-744, miR-4332, miR-92b-5p, miR-4334-5p	miR-185, miR-151-3p, miR-28-5p, miR-152, miR-151-5p, miR-1285, miR-20b, let-7e	0.19–0.29	4.11 × 10^−3^–2.03 × 10^−5^
HGB	miR-339-5p, miR-130a, miR-19b, miR-22-3p, miR-30e-5p, miR-744, miR-92b-5p, miR-199a-5p, miR-4334-5p, miR-4332	miR-151-3p, miR-28-5p, miR-28-3p, miR-185, miR-151-5p, miR-20b, let-7e, miR-146a-5p, miR-152, miR-195, miR-361-5p, miR-182, miR-421-3p, let-7d-5p, miR-150, miR-1285, miR-194b-5p, miR-30a-3p, miR-10a-5p, miR-128, let-7a, miR-98, miR-21, miR-7, miR-26a, let-7c, miR-214, let-7f, miR-100, miR-425-5p	0.19–0.37	4.85 × 10^−3^–3.38 × 10^−8^
RBC	miR-19b, miR-1307, miR-744, miR-92b-5p, miR-4332 miR-4334-5	miR-185, miR-152, miR-28-5p, miR-151-3p, miR-28-3p, miR-20b, let-7e, miR-151-5p, miR-195, miR-181b miR-146a-5p, miR-1285, miR-361-5p, miR-421-3p	0.19–0.37	4.19 × 10^−3^–2.88 × 10^−8^
MCV	miR-4334-5p	miR-27b-3p, miR-24-3p, miR-152, miR-28-3p	0.19–0.22	5.08 × 10^−3^–1.12 × 10^−3^
MCH	miR-145-5p, miR-193a-3p	miR-204	0.20–0.22	3.78 × 10^−3^–1.14 × 10^−3^
MCHC	miR-30d, miR-107, miR-4334-3p, miR-143-5p, miR-335, miR-22-3p, miR-29b, miR-4332, miR-339-5p, miR-19b, miR-497, miR-193a-3p, miR-324, miR-130a, miR-30e-5p, miR-199a-5p	miR-28-3p, miR-28-5p, miR-150, miR-151-3p, miR-128, miR-151-5p, miR-26a, miR-204, miR-20b, miR-195, miR-146a-5p, miR-421-3p, miR-10a-5p, miR-182, miR-23a, miR-30a-3p, miR-30e-3p, miR-98, miR-194b-5p, let-7d-5p, let-7e, miR-361-5p, let-7a, miR-139-5p, miR-193a-5p, miR-15b, miR-499-5p, miR-148b-3p, miR-100, miR-214, miR-24-3p, miR-18b, let-7c, miR-218b, miR-194a, miR-21, miR-152, let-7f, miR-125a, miR-185, miR-7, miR-342, miR-23b, miR-199b-3p, miR-215	0.20–0.42	4.46 × 10^−3^–6.31 × 10^−10^
MPV	miR-363, miR-20b		0.19–0.26	4.92 × 10^−3^–1.64 × 10^−4^
PLT		miR-497, miR-363	0.21–0.24	1.94 × 10^−3^–5.78 × 10^−4^
PCT		miR-497	0.24	4.18 × 10^−3^

**Figure 1.**
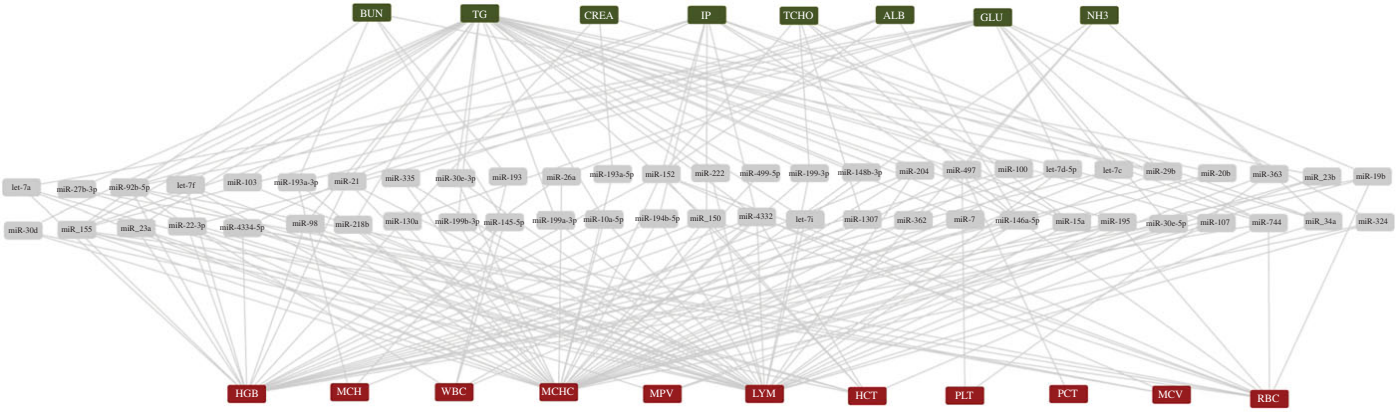
Common relationships between miRNAs, and biochemical and haematological traits. In total, 153 SSC-miRNA pairs were correlated with biochemical and haematological traits.

The expression levels of 254 miRNA species were correlated with biochemical and haematological traits. A total of 839 pairs of 254 miRNA species correlated with both metabolic and immune traits at FDR < 5% (electronic supplementary material, table S4). The most common pairs were found to include TG and other haematological traits (351 pairs). Figure 1 shows the 153 pairs of SSC–miRNA correlated with both biochemical and haematological traits.

### 2.2. Common traits correlated with microRNA and corresponding mRNA expression

Out of 6321 previously identified transcripts that were correlated with a minimum of one biochemical trait with *r* = |0.22–0.41| at a significance level of FDR < 1% [14]; in this study, we obtained those transcripts that were negatively correlated with miRNA expression and were correlated with the same biochemical traits. The transcripts were predicted as targets of miRNAs according to analyses with TargetScan and RNAhybrid. In total, 2843 combinations of miRNA and mRNA were determined to be linked to the same biochemical traits, shown in electronic supplementary material, table S5. Overall, eight biochemical traits (TG, TCHO, NH3, IP, GLU, CREP, BUN, ALB), 150 miRNA and 779 annotated transcripts were linked. Most links between traits and corresponding miRNA–mRNA pairs were identified for IP followed by TG, with 1637 and 755 pairs, respectively.

For haematological traits, 5387 transcripts from our previously study showing correlations with at least one of the traits at *r* = |0.22–0.48| and FDR < 1% were used for integrating with miRNA [14]. Using the same criteria, 3753 combinations between miRNA and mRNA linked to the same haematological traits were identified and are shown in electronic supplementary material, table S6. Overall, seven haematological traits (WBC, RBC, MCV, MCHC, LYM, HGB and HCT), 125 miRNA and 671 annotated transcripts were identified. Most links between traits and miRNA–mRNA pairs were identified for RBC (3178) followed by WBC (414).

### 2.3. Genetic regulation of microRNA transcripts expression-QTL

**Figure 2.**
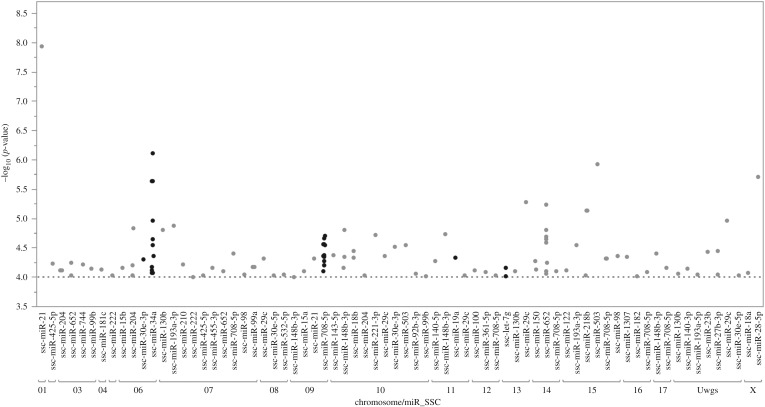
Manhattan plot of miR-eQTLs. Genome-wide association of SNPs and expression levels of miRNA located in pig genome Sscrofa 10.2. SNPs associated at a significance threshold (dotted line) *p*-value –log_10_ > 4 are shown. The miRNA and SNPs located on the same chromosomes are labelled as dark bold dots.

**Figure 3.**
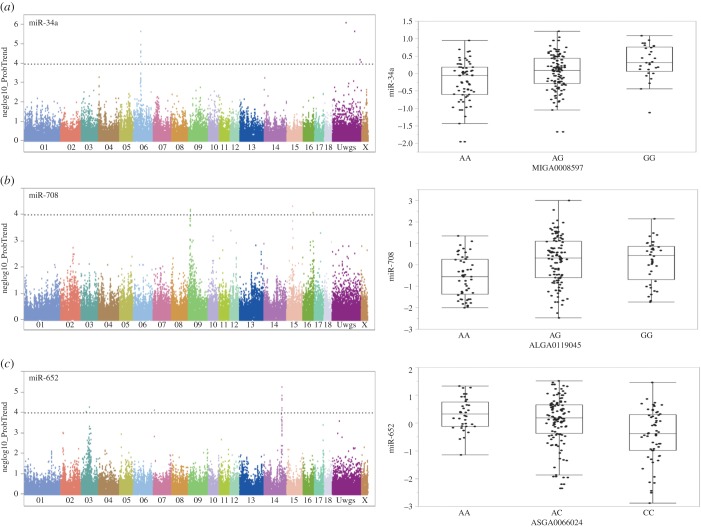
Genome-wide association of miRNA transcript levels. (*a*) Manhattan plot of *cis*-eQTL of miR-34a and associated SNP MIGA0008597 on pig chromosome 6 (SSC6). (*b*) Manhattan plot is the *cis*-eQTL of miR-708 together with the SNP genotype of ALGA0119045 on SSC9. (*c*) Manhattan plot is the eQTL of miR-652 and SNP genotype of ASGA0066024 on SSC14.

German Landrace pigs were genotyped and liver miRNA expression profiling was acquired using Affymetrix's Genechip miRNA 3.0 array. After quality control, 2736 probes (826 different miRNA species) from 209 individuals and 48 909 SNP markers were used for miRNA-eQTL analysis. A genome-wide association study between genotype and miRNA expression revealed 221 significant miRNA-eQTL that corresponded to 108 miRNA sequences and 164 SNPs at a thresholds of negative log 10 > 4 (electronic supplementary material, table S7). A total of 118 out of 221 significant miRNA-eQTL involved *Sus scrofa* mature miRNAs listed in the miRbase, whereas the other miRNA could be mapped on Sscrofa 10.2 by BLAST. The 118 miRNA-eQTL belonged to 45 miRNA species. Two miRNA (miR-708-5p and miR-34a) are highly associated with SNPs in surrounding regions (*cis*-eQTL). The other five miRNA transcripts were identified as locally regulated SNPs associated within the same chromosome of the probe-set/gene, including miR-30e, miR-19a, let-7 g, miR-4507 and miR-27d. The genetic regulated miRNAs miR-34a, miR-30e, miR-148-3p, miR-204, miR-181-5p, miR-143-5p and let-7g were also correlated with haematological and biochemical traits. Figure 2 shows the genetic regulation of SSC-miRNA across different pig chromosomes. Figure 3 depicts a Manhattan plot of miR-34a, miR-708 and miR-652 together with the top SNPs associated with their expression levels.

### 2.4. Common genotypes effect both mRNA and microRNA transcripts

Sixty-one SNPs were simultaneously associated with 29 miRNA species and 41 annotated transcripts (electronic supplementary material, table S8). The expression levels of 13 out of 29 miRNA correlated with biochemical traits or haematological traits. For example, miR-34a correlated with IP, and TCHO or miR-204, miR-15a, miR-16b correlated with TG. For haematological traits, the expression levels of miR-652 and miR-204 were found correlated with MCHC and the expression of miR-143 was found correlated with PLT. Eighteen out of 41 mRNA were previously assigned as *cis*-QTL [14]. Twelve out of 61 SNPs associated with miR-34a. Of note, miR-34a has not yet been annotated in the porcine genome. Human miR-34a is located on chromosome 1 at 9.15 Mb, a region syntenic to SSC6 position 64–65 Mb. Interestingly, SNPs associated with transcripts located on SSC6 at 55–65 Mb also associated with transcript levels of miR-34a. Notably, some of the SNPs and transcripts in this region are poorly annotated. The SNPs that significantly associated with miR-34a were also associated with *KIF1B* and *FBXO6*, which have a *cis*-eQTL. All nine SNPs associated with miR-708 located on SSC9 at 10–15 Mb were also associated with transcript levels of *POLD3, MOGAT2* and *CAPN5* in the same region, all of which are *cis*-eQTL. Three SNPs located on SSC6 associated with mRNA (CCDC30, RIMKLA, C3H1ORF50 and ST3GAL3) in the same regions (*cis*-mRNA) also associated with miR-204 on SSC1. SNPs (ASGA0036064) regulating the transcription of TTC8 in *cis* action also regulate miR-99a, miR-425-9p, miR-130b, miR-98, miR-140 in *trans*-action. Five out of seven SNPs found to regulate miR-652 were located on SSC14 and have a *cis*-mRNA.

**Figure 4.**
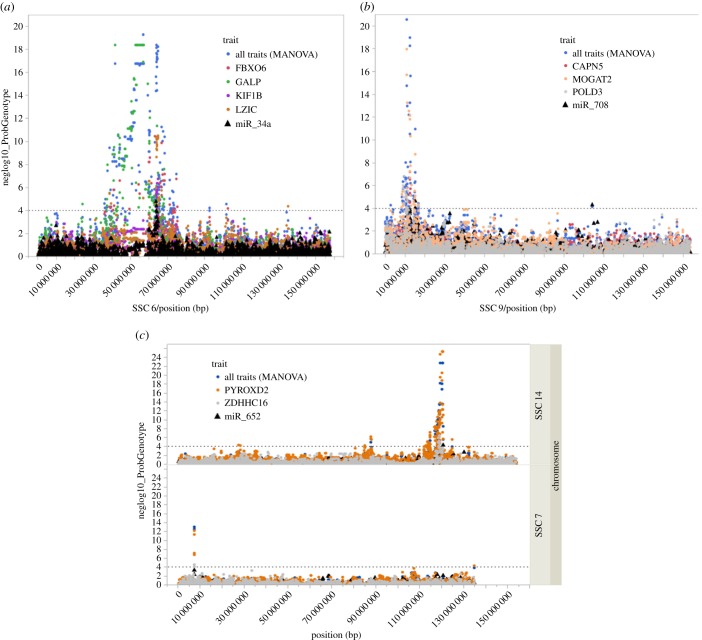
Pleiotropic association of miRNA and mRNA. Manhattan plots of pleiotropic associations between mRNA and miRNA expression. The pleiotropic association of these transcripts (all traits) were highly significant. (*a*) *cis*-eQTL of miR-34a and mRNA in vicinity on SSC 6; (*b*) *cis*-eQTL of miR-708 and mRNAs in the surrounding region on SSC9; (*c*) eQTL of miR-652 located on SSC14 and SSC7 associated with mRNA expression on the same chromosome. The *x*-axis indicates chromosome locations, *y*-axis shows −log10 of the *p*-values of MANOVA.

Genetic links between mRNA and miRNA were also shown using pleiotropic association analyses of miR-34a with GALP, LZIC, FBXO6 and KIF1B with 376 SNPs on SSC6 (FDR < 5%). miR-708 together with CAPN5, MOGAT2 and POLD3 were associated with 114 SNPs on SSC9 (FDR < 5%). miR-652 with PYROXD2 and ZDHHC16 with 71 SNPs on SSC14 and 2 SNPs on SSC7 (FDR < 5%) (figure 4).

## 3. Discussion

### 3.1. Trait-correlated hepatic microRNA expression is linked to metabolic and immune status

Holistic analysis of miRNA revealed not only trait-correlated expression but also a broad interaction between haematological and metabolic traits. For example, serum TG was correlated with miR-744, miR-199, miR-10, miR-23, miR-195 and miR-155, which were also correlated with erythrocyte-related traits (RBC, MCHC, HGB and MCV). Some of these interrelationships are evidenced by previous knowledge as exemplarily detailed here. This clearly indicates that our analyses provide biologically meaningful interactions. In fact, miR-155 plays a crucial role in several physiological processes including haematopoietic lineage differentiation, immunity and inflammation [23,24]. miR-155 is also involved in adipogenesis and lipid metabolism [25,26]. Overexpression of liver miR-155 in transgenic mice resulted in significantly reduced levels of serum total cholesterol, triglycerides (TG) and high-density lipoprotein (HDL) [26]. BUN is the major nitrogenous product of protein and amino acid catabolism and often used as an indicator of kidney and liver function. Interestingly, miR-155 is highly upregulated following ischaemic or toxic injury to the kidneys [27,28]. In this study, we show for the first time that there is a relationship between miR-155 expression and immune and metabolic parameters in healthy animals. We found that expression levels of miR-155 were highly negatively correlated with TG, ALB, BUN and IP. At the same time, miR-155 was also correlated with erythrocyte levels (RBC, HGB, HCT and MCHC) and LYM.

Similarly, miR-199a, miR-195, miR-10 and miR-23 expression were correlated with TG and haematological traits. Some of these miRNA are known biomarkers for hepatocellular carcinoma, including miR-199a [29] and miR-195 [30]. Insulin receptor (INSR) is a direct target of miR-195 [31]. Aberrant expression of miR-152 is related to hepatocellular carcinoma [32], as well as hepatic glycogenesis [33]. In our study, miR-152 was negatively correlated with TCHO and erythrocytes (RBC, MCHC).

We found that the expression of let-7 family mRNA negatively correlated with GLU levels, positively correlated with erythrocytes and negatively correlated with LYM. A previous study reported that overexpression of let-7 resulted in insulin resistance and impaired glucose tolerance, implying that the lin28/let-7 pathway regulates glucose metabolism [34]. In another study, it was reported that miRNA let-7 expression is regulated by glucose [35]. A number of studies have also demonstrated that let-7 miRNAs levels drop after infection, which in turn increases IL-10 mRNA [36]. It also regulates IL-13 expression and is associated with allergic airway inflammation [37], and suppresses adaptive immune responses, contributing to immune evasion of tumours [38].

Phosphorus (P) is a key element in numerous physiological processes, including energy transduction, cell signalling and regulation in metabolic pathways. We found that IP was correlated with a number of miRNAs including miR-34a and miR-194, which are both abundant in the liver. miR-34a is an obesity-associated miRNA that attenuates metabolic hormone function [39,40]. It is upregulated in the Doroc pig and Göttingen Minipig obesity model [41,42]. In other cases, miR-34a expression has been found to be increased in human patients with liver disease and in a rat liver fibrosis model [43,44]. miRNA-194 acts as a prognostic marker and inhibits proliferation in hepatocellular carcinoma by targeting MAP4K4 [45]. We detected additional miRNAs that were highly correlated with IP and erythrocytes including miR-1225 and miR-4334, whose functions remain unclear.

Blood ammonia (NH3) is an indicator of liver and kidney disease. Interestingly, we found that liver miRNA, including miR-138, miR-214 and miR-497, linked to NH3 and platelet traits (PCT and PLT). The functional link between platelets and blood NH3 mediated by miRNA is unknown. Most of these miRNAs are reported as biomarkers in renal tumourigenesis and hepatocellular carcinoma [46,47]. miR-214 appears to participate in the development of hepatic fibrosis [48], while miR-497 regulates cell proliferation in hepatocellular carcinoma [49]. While links between miRNAs and specific traits (mostly pathologic conditions) have been shown, we provide evidence that many miRNAs represent common links between metabolic and haematological and immune-related traits.

### 3.2. Common traits correlated with microRNA and mRNA

Our previous study identified liver transcripts related to cellular and molecular processes and established a comprehensive view of hepatic gene activity linked to metabolic and immune traits in pigs [14]. The fact that thousands of genes have potential binding sites for miRNAs and single miRNAs can simultaneously target hundreds of genes [50,51] makes the data analysis challenging even with the use of prediction software and criteria. To determine the relationship between identified miRNAs and their targets, we used previous data taken from the same samples. We previously identified 6321 transcripts that were correlated with a minimum of one biochemical trait with *r* = |0.22–0.41| at a significance level of FDR < 1% [14]. In order to focus our study, after target prediction using miRNA seed sequence analysis, only transcripts (mRNA and miRNA) correlated with the same traits and that were negatively correlated with each other were analysed. For instance, BUN, a biomarker for kidney and liver function, was correlated with miR-145, miR-18, miR-29a and miR-671. These miRNAs were related to transcripts including *SAP30 L, FZD4, BMPR2, LEPR, SCD, ATP1A1, CXCL12, GC, LEPR, SLC16A2, DPP4, SULT2A1, PHB, UBR1* and *DPP4*, which were also correlated with BUN and are involved in adipogenesis or lipid metabolism pathways. Interestingly, as previously reported, BUN is correlated with fat traits [14].

MiRNAs including let-7, miR-1224 and miR-2288 that correlated with glucose levels were also correlated with target mRNAs belonging to functions of carbohydrate metabolism, including *ACACA, ATXN2, FASN, ESR1, FUT8, SCD, CTH, GPR39, ITGB3, MLXIPL, BCL2, RGL1, MTAP, PGM2* and *SLC16A6*. As discussed above, the let-7 family plays a significant role in glucose metabolism; this study shows the complex levels of regulation.

Additional relationships were identified between haematological traits, miR-145 and transcripts of acute phase response signalling like *SOCS3* and *STAT3*. Targets of miR-1915 that correlated with RBC were found to be enriched in LXR/RXR activation. Interestingly, Aconitase 1 (ACO1) is involved in the control of iron homeostasis [52] and superoxide dismutase 1 (SOD1) has a significant role on the lifespan and quantity of red blood cells in peripheral blood [53]; both of these were predicted targets of miR-1777b [14]. MiR-1777b was highly correlated with RBC (*r* = 0.397, *p* = 3.97 × 10^−9^). This miRNA is still not annotated in pigs; however, using BLAST, we found a sequence identical to miR-1777b located on SSC6 at 827723 bp.

The complex miRNA–mRNA regulatory networks identified by this study may contribute to fine-tuning liver gene expression and may have a significant impact on metabolic and immune traits.

### 3.3. Genetic regulation of microRNA transcripts

Genetic polymorphisms that affect miRNA levels can contribute to a wide range of target genes and phenotypes. We obtained SNP genotypes and assessed whole-genome miRNA expression in the liver to identify genetic variations that influence miRNA and mRNA targets related to immune and metabolic traits. A low number of *cis*-miRNA-eQTL was also reported in a study of primary fibroblasts derived from the umbilical cord [54]. Other reports of genetic regulation of miRNA expression in the skin revealed 42 eQTL for 38 miRNAs, all of which were *trans*-eQTL [20]. *Trans*-only miRNA eQTL in the liver have also been reported [21]. Other studies report a limited number of miRNA eQTLs in adipose tissue [55] and dendritic cells [18]. By contrast, an eQTL study of whole blood miRNAs from a large sample size revealed about 27% of miRNA with *cis* effects [19]. Compared with our previous study with eQTL of mRNA from the same samples, we found that about 75% of mRNA have *cis* effects [14]. In this study, we found miR-708 as *cis*-eQTL with the windows smaller than 1 Mb and the transcripts in the surrounding region also regulated with the same SNPs. Based on the synteny between the human and pig genome and the discovery of other transcripts in this region regulated with the same SNPs, we expected miR-34a to be *cis*-eQTL. Five other miR (miR-30e, miR-19a, miR-4507, miR-27d and let-7 g) were associated with SNPs on the same chromosome. Other miRNA (like miR-652) are still unmapped (NW_003539729) in the pig chromosome. Most of the miRNA-eQTL in this study were identified as *trans*-eQTL. We identified a number of miRNA that were correlated with haematological and biochemical traits. Since miRNAs can target many mRNAs, the effect of genetic variants on miRNA expression can play an important role in complex phenotypes mediated by various target genes.

### 3.4. Shared genotype effects on mRNA and microRNA transcripts

SNPs located at the same region as its regulated transcripts were identified as *cis*-eQTL. These SNPs can regulate many transcripts in the vicinity. Subsequently, miRNA eQTLs will be enriched for eQTLs of host genes as shown in our study. Interestingly, most of the SNPs regulating mRNAs that were *cis*-eQTL at the same time also regulate miRNA. We found that SNPs around miR-708 and miR-34a were also associated with the mRNA transcripts in the vicinity. Recently, the majority of shared eQTLs of miRNAs and mRNAs were found to have different effects on miRNA or mRNA transcription, suggesting an independent regulation of miRNAs and mRNAs [56]. miR-34a was negatively correlated with IP (*r* = 0.35, *p* = 2.26 × 10^−7^). The same SNPs that regulated miRNA-34a also regulated mRNAs including FBXO6, KIF1B, GALP, LZIC, LOC100516739 and NMNAT1. In our previous study, we found that the expression pattern of Galanin-like peptide (GALP) was also negatively correlated with IP (*r* = 0.28, *p* = 7.9 × 10^−7^). GALP is a neuropeptide regulating feeding behaviour and is involved in energy metabolism [57,58]. Interestingly, high-phosphorus diets enhance energy metabolism through the utilization of free fatty acids released via lipolysis of white adipose tissue [59]. Accordingly, our previous study showed that miR-34a and miR-708 are upregulated in Duroc pigs, which are more obese than Pietrain [42]. This information links miR-34a to IP and TCHO, providing evidence of a functional link between IP, energy metabolism, genetic regulation of miRNA 34a and GALP.

We found that SNPs located on SSC9 associated with Aquaporins (AQP11) and miR-15a/16a. Using target site prediction, AQP11 was identified as a target of miR-15a/16 in many species including human, rat and mouse. Aquaporins are membrane water/glycerol channels involved in many physiological processes including circulation of glycerol and adipocyte metabolism [60]. In the present study, miR-15a was also found to be negatively correlated with TG and TCHO. miR-15a, however, has not been located in the pig genome. Genetic regulate miR-652 also regulate PYROXD2 and ZDHHC16 located on SSC14 and SSC7. Our previously study determined *PYROXD2* and *ZDHHC16* as *cis*-eQTL, but miR-652 has not been located in the pig genome.

The analysis of miRNA transcript profiles together with information on their genetic regulation provide a new resource for understanding the genotype–phenotype mapping associated with hepatic gene expression targets and the physiological processes related to haematological, immune and metabolic traits. Our findings suggest a substantial overlap of the genetics of miRNA and mRNA. In fact, we demonstrated that SNPs show pleiotropic effects by simultaneously affecting miRNA and mRNA expression and thus build regulatory networks influencing complex traits. Our analyses of trait-correlated miRNA and eQTL detection complement genome-wide association studies (GWAS) of immune and metabolic traits. The study supports the notion that miRNA can be used as biomarkers by showing links between liver miRNA and mRNA expression and plasma haematological, biochemical and clinical–chemical parameters.

## 4. Material and methods

### 4.1. Animals and sample collection

Animal care and tissue collection procedures followed the guidelines of the German Law of Animal Protection. The experimental protocol was approved by the Animal Care Committee of the Leibniz Institute for Farm Animal Biology. Performance-tested pigs from commercial herds of German Landrace pig were used for GWAS of liver miRNA (*n* = 209). Liver and blood samples were collected from pigs at an average age of 170 days at the experimental slaughter facility of the Leibniz Institute for Farm Animal Biology. Veterinary inspection of the carcasses and organs after slaughter confirmed a lack of any impairments, disease symptoms or pathological signs to avoid any bias of blood phenotypes. Blood serum was prepared by centrifugation and haematological and biochemical traits were determined using automated analyser devices (ABX Pentra 60 HORIBA, Montpellier, France; Fuji DriChem 4000i, FujiFilm, Minato, Japan; electronic supplementary material, table S1).

Genotyping was performed using the PorcineSNP60 BeadChip (Illumina Inc., San Diego, CA, USA) as per the manufacturer's SNP Infinium HD assay protocol. In brief, 200 ng of DNA was amplified, fragmented and hybridized to the PorcineSNP60 BeadChip containing 62163 locus-specific 50-mers covalently linked to beads distributed on the microarray surface. Single-base extension of captured oligos incorporated labels detected by Illumina iScan and images were subsequently converted to intensity data. Intensity data were normalized and assigned a cluster position, genotype and quality score with GenomeStudio software (Illumina Inc.). Samples with call rates less than 99% were removed. Markers with low minor-allele frequency (less than 5%) were also excluded. Markers that strongly deviated from the Hardy–Weinberg equilibrium (*p* < 0.0001) were also excluded. The average call rate for all samples was 99.8% ± 0.2. The markers of the 60 K chip were mapped to the porcine reference genome using Sscrofa 10.2 (Ensembl; downloaded from NCBI, http://www.ncbi.nlm.nih.gov).

### 4.2. RNA isolation and microRNA microarray analysis

Small RNAs were isolated and enriched from liver using an miReasy Mini kit and an RNeasy MinElute Cleanup kit (Qiagen, Hilden, Germany) according to the manufacturer's protocols. The quality and quantity of small RNA were assessed with an Agilent 2100 Bioanalyser (Agilent, Santa Clara, CA, USA) using an Agilent small RNA kit. miRNA expression profiling was performed using the GeneChip miRNA 3.0 array (Affymetrix, Santa Clara, CA, USA) according to the manufacturer's recommendations. Affymetrix Gene Chip Micro 3.0 Array provides 100% miRBase v17 coverage (www.mirbase.org). The microarray contains 12187 unique sequences for the detection of mature miRNAs assigned to *S. scrofa* and other species including human, providing a highly redundant expression analysis platform because many miRNA probes of various species are identical, reflecting the high degree of conservation of miRNAs. Small RNAs (200 ng) were used for sample preparation with a FlashTag Biotin RNA labelling kit for Affymetrix GeneChip miRNA arrays (Genisphere, Hatfield, PA, USA). Labelled RNA was then hybridized for 16 h to the miRNA arrays according to the manufacturer's recommendations, washed and stained in a Fluidics Station 450, and scanned on a G3000 GeneArray Scanner (Affymetrix). Robust multi-array average (RMA) background correction, log_2_ transformations and quantile normalization methods implemented in JMP Genomics 6 (SAS Institute, Cary, NC, USA) were performed.

### 4.3. Bioinformatic analysis

All miRNA probe-set sequences were used to blast against miRBase 21 (SSC miRNA) and pig genome (Sscrofa 10.2). All miRNA species that were not available in the SSC miR-database or could not be mapped in pig genome were excluded. A total of 2736 probe-sets (826 different miRNA species) passed the filtering and were used for further analyses. miRNA microarray data were deposited in the Gene Expression Omnibus public repository (GEO accession numbers: GSE97274, GSM2560488–GSM2560696). TargetScan was used first to detect predicted target genes based on seed complementarity on both 3'- and 5′-UTR and coding sequences of the porcine mRNA sequences (Sscrofa 10.2) and miRNA species from our study. Further, RNAhybrid software (http://bibiserv.techfak.uni-bielefeld.de/rnahybrid) was used for direct prediction of multiple energetically favourable potential binding sites (energy cut-off = −25 kcal mol^−1^). Our previous mRNA expression data were integrated with miRNA data from the same samples. We further identified mRNA–miRNA pairs that were negatively correlated (FDR < 5%) with each other and that correlated with the same traits (FDR < 5%).

### 4.4. Data pre-processing and statistical analysis

After quality control and filtering, the expression data were further pre-processed to account for systemic effects. Mixed-model analyses of variance using JMP Genomics (SAS Institute, Cary, NC, USA) were used to adjust for the effect. The genetic similarity matrix between individuals was first computed as identity-by-descent of each pair for the *k*-matrix and used (considered) as a random effect. For control of population stratification, top principal components (PCs) explaining a variation of more than 1% were considered as covariates. In total, 17 PCs were included as covariates. Gender was used as a fixed effect, slaughter day was used as a random effect and carcass weight was considered as a covariate. The residuals were retained for further analysis.

eQTL analyses were conducted using the R package Matrix eQTL [61]. Matrix eQTL tests for association between each SNP and residual transcripts levels were assessed by modelling the additive effects of genotypes in a least square model [61]. Matrix eQTL performs a separate test for each gene–SNP pair and corrects for multiple comparisons by calculating the FDR [62]). Annotation and localization of SNP sites and probe-sets (Sscrofa 10.2) allowed for discrimination of *cis*- and *trans*-regulation. We defined an eQTL as *cis* if an associated SNP was located within an area less than 1 Mb from the probe-set/gene.

The common regions linking mRNA and miRNA were analysed by pleiotropic association. Pleiotropic association analyses were performed by MANOVA (multivariate analysis of variance) between miRNA and mRNA transcripts levels and genetic marker data.

The correlation of miRNA transcript levels with haematological and biochemical traits were estimated as Spearman coefficients and corrected for multiple comparisons by calculating the FDR [62].

## Supplementary Material

Table S1

## Supplementary Material

Table S2

## Supplementary Material

Table S3

## Supplementary Material

Table S4

## Supplementary Material

Table S5

## Supplementary Material

Table S6

## Supplementary Material

Table S7

## Supplementary Material

Table S8

## Data accessibility

The availability of expression data are in the Gene Expression Omnibus public repository with the GEO accession number GSE97274, GSM2560488–GSM2560696.
