# Supplementary material for: Genetic architecture and regulatory impact on hepatic microRNA expression linked to immune and metabolic traits
Source: Open Biol. 2017 Nov 8;7(11):170101. doi: 10.1098/rsob.170101 (PMC5717336; doi:10.1098/rsob.170101)
Supplement: Table S1 [file rsob170101supp1.docx]

**Electronic supplementary material**

**Siriluck Ponsuksili, Nares Trakooljul, Frieder Hadlich, Fiete Haack, Eduard Murani, and Klaus Wimmers**

**Genetic architecture and regulatory impact on hepatic microRNA expression linked to immune and metabolic traits**

**Open Biology**

**DOI: 10.1098/rsob.2016**

Table S1: Definitions of traits, number of samples, means, standard deviations for each of the haematological and biochemical traits.

| Traits | Definitions of traits | | | | Mean±SD (N=209) | |
| --- | --- | --- | --- | --- | --- | --- |
| WBC (10³/mm³)  LYM (#)  RBC (106 /mm³)  HGB (g/dl)  HCT (%)  MCV (µm³)  MCH(pg)  MCHC (g/dl)  RDW (%)  PLT (10³/mm³)  MPV (µm³)  PCT(%) | | | White blood cell count  Lymphocytes count  Red blood cell count  Hemoglobin concentration  haematocrit level  Mean Corpuscular Volume  Mean Corpuscular Haemoglobin  Mean Corpuscular Haemoglobin Concentration  Red Distribution Width  Platelets  Mean Platelet Volume  Plateletcrit | 20.16±4.8  8.42±1.9  8.07±0.7  13.60±1.2  43.44±3.2  53.89±2.8  16.89±1.2  31.33±1.4  16.07±1.4  303.20±68.9  7.51±0.5  0.23±0.05 | |  |
| ALB (g/dl)  NH3 (µg/dl)  BUN (mg/dl)  TCHO (mg/dl)  TG (mg/dl)  GLU (mg/dl)  IP (mg/dl)  CREA (mg/dl) | | Albumin concentration  Ammonia nitrogen concentration  Blood urea nitrogen concentration  Total cholesterol  Triglyceride concentration  Glucose concentration  Inorganic phosphorus concentration  Creatinine concentration | | 4.37±0.3  83.22±21.3  14.66±3.6  86.97±12.3  41.50±13.9  131.98±20.9  8.50±1.2  1.34±0.2 | |  |
